# State Work with Infectious Diseases of Animals1Presented at the annual meeting of the State Agricultural Society, at St. Paul, January 10 and 11, 1900.

**Published:** 1900-05

**Authors:** M. H. Reynolds

**Affiliations:** St. Anthony Park, Minn.


### STATE WORK WITH INFECTIOUS DISEASES OF ANIMALS.[1]

By M. H. Reynolds, M.D., V.M.,
ST. ANTHONY PARK, MINN.

(Continued from page 157, March, 1900.)

*Hog-cholera.* I have been somewhat disappointed with the progress we have made in the hog-cholera work during 1899, and I feel that we are at a critical point in this work. Very satisfactory progress was made in 1897 and 1898, as I will soon show in a brief table. The difficulty this year has been to secure prompt reports of outbreaks, and this difficulty is due to an outcropping

[1] Presented at the annual meeting of the State Agricultural Society, at St. Paul, January 10 and 11, 1900.

of natural human selfishness. Unless the State Board can secure prompt reports, the work of further reducing the infected area and financial losses will be greatly hindered.

During the past quarter thirty-nine townships in nineteen counties have been reported as infected with hog-cholera.

The following table gives a reasonably accurate survey of the situation during 1897, 1898, and 1899. The number of townships and financial losses for 1898 and 1899 should probably be somewhat larger. Please bear in mind that this work did not begin until 1897, and that forty-three counties were then infected. It is not difficult to check a small outbreak of hog-cholera and completely eradicate the disease, but forty-three infected counties to deal with in a State as large as Minnesota is a very different proposition.

| Year. | Counties infected. | Townships infected. | Loss . |
|---|---|---|---|
| 1896 | 43 | — | $1,000,000 |
| 1897 | 41 | 254 | 487,500 |
| 1898 | 32 | 93 | 325,000 |
| 1899 | 32 | 75 | 252,000 |

I wish to explain again that this table is not presented as being absolutely accurate, but it is as accurate as existing information allows. The relative proportion of losses and townships infected is probably very nearly correct. Other natural conditions have had something to do with that decrease in the infected area and the financial losses. It is difficult to estimate just what proportion should be credited to the work of the State Board and what to natural conditions, but it is evident tnat these natural conditions do not alone account for the improved situation. The following is submitted as showing the needs of general information concerning hog-cholera and the necessity of vigorous work. Hog-cholera appeared in a certain township of Stearns County during the early summer. The township chairman called on the owner soon afterward and quarantined the hogs and pens, giving instructions as to what should be done. The chairman considered this owner a good citizen, and did not watch things as closely as he would otherwise have done. We have since learned that this farmer lost quite a lot of hogs. They were left unburied, although the law expressly orders to the contrary. The outbreak in this township has since been around this man's farm in all directions, and has apparently been spread largely by dogs.

There are two adjoining townships in one of our most southern counties where hog-cholera appeared last June. The chairman did nothing to check its spread, and the outbreak became very general. They did not even obey the most explicit directions of the law. Three adjoining townships received infections from these two, but infected places were quarantined, and in no case did the disease spread beyond the first quarantine. The two generally infected townships reaped a disastrous harvest. This would not be so bad if they had not been a constant source of danger for adjoining townships where the chairmen and farmers were law-abiding.

An interesting situation developed in connection with an outbreak in Polk and Red Lake Counties. During the course of our work last season we learned that hogs had died on a certain farm during the previous fall. Dead hogs were thrown into Red Lake River in the northern part of Gentilly Township. Red Lake River is reported as being a very sluggish stream, and in the early spring outbreaks appeared on a series of farms along this river, down stream. The disease was then carried from these farms to the surrounding country, and a very serious outbreak of hog-cholera was the result; and yet intelligent people say that hog-cholera spreads in the air and in a great variety of mysterious ways, and hog-cholera is just hog-cholera, and there is no use in attempting to do anything with it. The outbreak just referred to had its plain origin when the careless farmer threw dead hogs into the sluggish stream.

It is very probable that there have been few outbreaks of hog-cholera in Minnesota where the method of spread was not simple and easily understood if we could only get the facts.

Mr. ———, of Greenville Township, Dakota County, assisted a friend to load a car of hogs, and during this work he was in the stock-car. After finishing the work he went home and took care of his own hogs. In about two weeks cholera broke out in his herd, with no other cases in the vicinity, and yet he only walked around in the stock-car and some railroad shipping pens.

Mr. G. went to Shakopee and attended a street fair. Bought seven young pigs. Took them home. In a few days these pigs were sick, some of them dying. In about two weeks the rest of his hogs were taken sick, and they all died. A neighbor helped Mr. G. move the pigs over from the wagon in which they were brought to the sale into Mr. G.'s wagon. He then went home, and in about two weeks his hogs were dying with cholera.

Not very long ago a farmer living near Morgan, Redwood County, shipped in some hogs from Olmsted County, and, by the way, contrary to the regulations of the State Board of Health.

These imported hogs infected those previously on the place, and resulted in an outbreak of cholera.

A farmer living near Sleepy Eye sold some hogs to a buyer at that place. The hogs were placed in the shipping pens, and, contrary to the regulations of the State Board of Health, sold them to another, farmer who took them back to the country. These hogs were free from the disease when the first sale was made. No cholera had existed in the neighborhood from which they came. After the usual period of about two weeks they became sick and were dying with cholera. Result: another outbreak of cholera. It is very evident that these hogs were infected in the railroad shipping pens.

Suppose a case: Hog-cholera appears in a previously uninfected district. The outbreak is confined to two or three farms. In a reasonably intelligent and law-abiding neighborhood this outbreak can be quarantined with reasonable certainty of complete success, providing the chairmen posts quarantine placards, visits the neighbors, gives all the information at hand concerning the nature of the disease, how it spreads, etc., and urges owners whose hogs are not infected to post quarantine cards, etc.

But suppose the chairman takes no interest; suppose the owner leaves carcasses of hogs unburied after a representative of the State Board of Health has left. Dogs are numerous. Neighbors refuse to accept suggestions concerning dogs and other sources of spread; visitors pay no attention to the notice cards—quarantine is a failure. The disease spreads rapidly, not necessarily because the theory of quarantine is wrong, but merely because of the listlessness and general uselessness of the chairman and the stubbornness and selfishness of the owner and the indifference of the neighbors. "Render to Cæsar the things that are Cæsar's."

That it is possible to maintain successful farm quarantine for hog-cholera has been demonstrated over and over again during the past three years. Whether individual farm quarantine is successful or not depends largely upon the township supervisors and the individual farmers. The mere fact that individual farm quarantine has not been entirely successful in many townships does not contradict this statement. The thing for the farmers of Minnesota to do is to let go of this idea of finding a medical cure for hog-cholera and undertake an intelligent prevention. It is probable that a satisfactory serum for vaccination will be developed in the near future, but that alone will not solve the problem by any means or do away with the necessity of sanitary measures.

The following extracts from the "Circular of Information for Local Health Officers," together with the "Hog-cholera" quarantine and the owners' "notice" cards, will give some information concerning the present methods of dealing with this disease.

When hogs begin to sicken and die during the prevalence of hog-cholera the disease should be reported to the local health officer or chairman of the town board, and quarantine should be established at once. It is a simple matter to release quarantine, and should it be proven that the disease is not hog-cholera, no harm has been done by such quarantine.

All health officers and acting health officers are therefore instructed to see that suspicious outbreaks of disease among hogs are properly quarantined.

The health officer should explain to the owner or keeper the nature and conditions of quarantine, and see that the conditions are rigidly enforced until quarantine is released.

Hog-cholera is often spread by water in small streams and lakes, and for this reason hogs must not be buried near any such lake or water-course.

Dogs and poultry should not be allowed access to yards or pens where hogs are confined during the hog-cholera season, and pigeons and crows should be shot or otherwise frightened away because of danger that they may spread the disease.

The health officer or inspector should always wear overalls and overshoes or rubbers when going among diseased hogs. These overalls and overshoes or rubbers should always be kept at a safe distance from healthy hogs and from other agents which might convey the disease.

Quarantine cards must not be removed until six months after the last hog has died or recovered, and the premises disinfected in a way satisfactory to the local board of health.

Farmers should be urged to dispose of marketable hogs for slaughter as soon as suspected hog-cholera appears in a neighborhood.

Neighbors on whose farms the disease has not yet appeared should never be allowed to help haul the hogs from infected farms, as there is great danger that the disease will be spread from farm to farm by such action.

*Tuberculosis.* I wish to make a special effort at this meeting to interest members of this association in the subject of "Bovine Tuberculosis," particularly as affecting the cattle-breeding interests. The problem is a very large one and extremely difficult. There is no possibility of question but that tuberculosis prevails among pure bred cattle. This needs no further demonstration; neither is there any question as to the reliability of tuberculin as a means of diagnosis. This has also been demonstrated beyond possibility of reasonable question. It is practically settled that the germ causing bovine tuberculosis and the germ causing human tuberculosis are identical as to species and differ only as modified by slightly different environments in different animal bodies. These and a host of other questions need not trouble us. The difficult question is,

What to do about it? What steps to take and how to take them in order that the spread of tuberculosis among pure bred cattle can be lessened or controlled without at the same time causing the breeder great financial losses and endangering the cattle-breeding interests of the State, which it is our business to foster rather than injure. I will have some suggestions to make later concerning this problem.

(To be continued.)

